# First person – Haimeng Lyu

**DOI:** 10.1242/dmm.052019

**Published:** 2024-08-14

## Abstract

First Person is a series of interviews with the first authors of a selection of papers published in Disease Models & Mechanisms, helping researchers promote themselves alongside their papers. Haimeng Lyu is first author on ‘ Functional distinction in oncogenic Ras variant activity in *Caenorhabditis elegans*’, published in DMM. Haimeng conducted the research described in this article while a Research Assistant in Helen Chamberlin's lab at The Ohio State University, Columbus, Ohio. She is now a Research Senior Technician in the lab of Wenjing Sun (Department of Neuroscience, Columbus), investigating mechanisms that mediate myelin development.


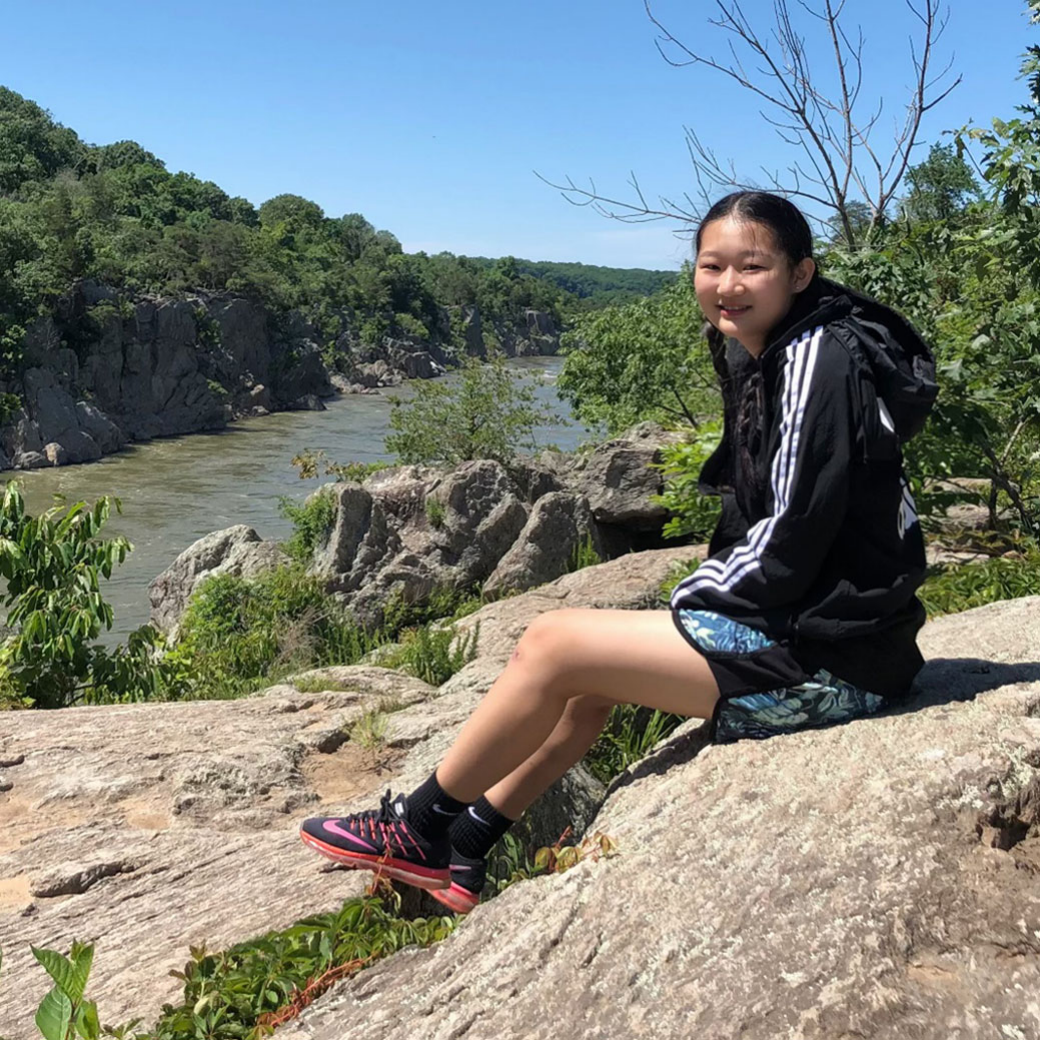


**Haimeng Lyu**

**Who or what inspired you to become a scientist?**

I spent almost half of my childhood in the hospital as close members of my family were diagnosed – seemingly one by one – with serious diseases. Watching my grandfather, to whom I was very close, suffering not only because of the disease itself but also from the brutal side effects of chemotherapy and surgery tormented me. I wanted to help him but did not know how. Watching doctors diagnose patients based on scientific principles, rely on research findings to advance medical knowledge and improve patient care inspired me to dedicate my studies to the sciences. The more I learned from my science courses, the more curious I became about the unknown. This motivated in me the desire to solve complex unknowns and sparked my interest in becoming a scientist.

**What is the main question or challenge in disease biology you are addressing in this paper? How did you go about investigating your question or challenge?**

In our paper we focused on *Ras* genes, a family of significant oncogenes commonly mutated in cancer. Since only a few studies have been conducted to assess how distinct Ras variants influence and respond to their embedded cellular networks, we aimed to uncover these Ras variants by using a simple vulva development assay in *C. elegans*. With major oncogenic mutations occurring at amino acids G12, G13 and Q61, we introduced substitutions of these residues using CRISPR-mediated genome editing and a Mos1-mediated transposon landing site. We evaluated and grouped these mutations based on phenotypic difference, sensitivity to gene dosage and response to non-autonomous genetic modulators. Overall, we found that *C. elegans* Ras genes exhibit distinct properties and show different effects in response to extracellular modulators.

**How would you explain the main findings of your paper to non-scientific family and friends?**

Ras genes are sequences of the genome altered frequently in many cancers, including pancreatic, colorectal and lung cancer. These cancer-causing genetic Ras variants present differently in different cancer types and demonstrate variable sensitivities in response to pharmacological treatment. One aspect of these distinct and poorly understood variants is their cellular interactions. By studying and identifying the distinct Ras gene substitution changes *in vivo* using the roundworm model *C. elegans*, we uncovered a set of criteria that can be used to group different Ras variants both morphologically and functionally. This is important, as it helps us to learn more about the distinction of Ras variants and explore new possibilities to target each variant more effectively based on its properties.

**What are the potential implications of these results for disease biology and the possible impact on patients?**

Since Ras genes oncogenes are frequently mutated in many cancers, understanding how these distinct variants influence and respond to cellular networks is crucial. By identifying the distinct functional groups of these Ras variants, we were able to distinguish them on the basis of phenotype, gene dosage sensitivity and response to non-autonomous genetic modulators. Based on the phenotypic differences, we proposed that Ras variants can be screened using simple morphological criteria followed by functional distinctions. Furthermore, we demonstrated that oncogenic mutations display unique sensitivity to external modifiers, thereby providing an experimental system to discover new genes that mediate or modulate Ras activity. In the future, all this might contribute to developing more effective pharmacological treatments to target oncogenic Ras variants.

**Figure:**
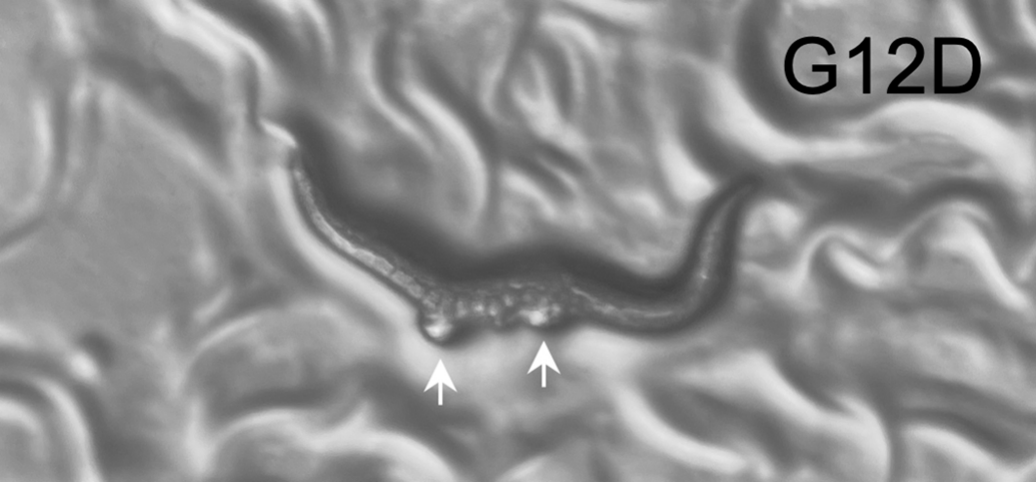
**Introduction of G12D mutation orthologous to human oncogenic Ras variants produces the Multivulva phenotype in *C. elegans*.** The animal is oriented with anterior to the right and ventral side down. Arrows indicate ventral bumps.

**Why did you choose DMM for your paper?**

DMM is a peer-reviewed journal dedicated to advancing the understanding of mechanisms, assessment and treatment of human diseases by using trailblazing model systems. In our paper, we used a *C. elegans* model to search for novel participants in the complex interplay between different Ras genotypes and cell interactions, which aligns precisely with the journal's objectives. In addition, we believe that DMM provides a great platform for researchers to review and provide useful insights regarding our findings, and promotes further understanding and advancement in our field of research.

**Given your current role, what challenges do you face and what changes could improve the professional lives of other scientists in this role?**

As a person still in the early stage of a research career, I sometimes struggle to find direction and resources for guidance. There are many possible paths to take in this field, which can be overwhelming and intimidating. Finding where and how to start is difficult with my limited experience. I believe platforms, such as those including first author interviews, are a great way to engage with other scientists, learn about their experiences and reflect on oneself. I think an expansion of such platforms for young scientists, like informal seminars and meetings to discuss opportunities for early researchers would be very helpful.

**What's next for you?**

I am currently applying to MCDB PhD programs starting in autumn 2025. In the future, I hope to work on projects relating to gene therapy or genetic engineering to treat cancer. I am very excited about the next stage of my career, which is attending graduate school, especially after learning so much about scientific research during my time as a research technician. I realized that research extends far beyond applying theoretical knowledge, and that troubleshooting is a frustrating but essential part of research. This has allowed me to accept that, instead of thinking an experiment has failed, I have eliminated another incorrect answer. This mindset has helped me through many difficulties and, I believe, will help me in graduate school in the future.

**Tell us something interesting about yourself that wouldn't be on your CV**

Aside from working and studying, I love horseback riding and snowboarding. These are important activities in my life to help me cope with stress and stay focused. I believe work–life balance is crucial to help maintain productivity and effectiveness, which is why these hobbies are invaluable in my life.
